# Cardiovascular safety of psychedelic medicine: current status and future directions

**DOI:** 10.1007/s43440-023-00539-4

**Published:** 2023-10-24

**Authors:** Agnieszka Wsół

**Affiliations:** Chair and Department of Experimental and Clinical Physiology, Laboratory of Centre for Preclinical Research, Medical University of Warsaw, Banacha 1B, 02-097 Warsaw, Poland

**Keywords:** Psychedelics, Cardiovascular, Psilocybin, LSD

## Abstract

Psychedelics are powerful psychoactive substances that alter perception and mood processes. Their effectiveness in the treatment of psychiatric diseases was known before their prohibition. An increasing number of recent studies, due to the indisputable resurgence of serotonergic hallucinogens, have shown their efficacy in alleviating depression, anxiety, substance abuse therapies, and existential distress treatment in patients facing life-threatening illness. Psychedelics are generally considered to be physiologically safe with low toxicity and low addictive potential. However, their agonism at serotonergic receptors should be considered in the context of possible serotonin-related cardiotoxicity (5-HT2A/2B and 5-HT4 receptors), influence on platelet aggregation (5-HT2A receptor), and their proarrhythmic potential. The use of psychedelics has also been associated with significant sympathomimetic effects in both experimental and clinical studies. Therefore, the present review aims to provide a critical discussion of the cardiovascular safety of psilocybin, d-lysergic acid diethylamide (LSD), N,N-dimethyltryptamine, ayahuasca, and mescaline, based on the results of experimental research and clinical trials in humans. Experimental studies provide inconsistent information on the potential cardiovascular effects and toxicity of psychedelics. Data from clinical trials point to the relative cardiovascular safety of psychedelic-assisted therapies in the population of “healthy” volunteers. However, there is insufficient evidence from therapies carried out with microdoses of psychedelics, and there is still a lack of data on the safety of psychedelics in the population of patients with cardiovascular disease. Therefore, the exact determination of the cardiovascular safety of psychedelic therapies (especially long-term therapies) requires further research.

## Introduction

Psychedelics are a class of psychoactive substances, whose “acute” effects resulting primarily from serotonergic 5-HT2A receptors agonism cover a wide spectrum of behavioral, psychological, and physiological effects [1–3].

A range of symptoms, particularly those affecting the state of consciousness, caused by plants or plant-derived substances and fungi containing psychedelic substances, have led to their use for millennia in religious practices, tribal rituals, and also for the treatment of fever and rheumatic pain [4–6]. The modern era of psychedelic research did not begin with the synthesis of a new substance in this group, d-lysergic acid diethylamide (LSD) in 1938, but with the accidental discovery of its effects by Albert Hoffmann on 19 April, 1943, (known as Bike Day) [7]. This opened the door to extensive research into the use of the LSD molecule (then produced under the trade name *Delysid*) in the treatment of mental illnesses, such as: mood disorders, anxiety, and acute stress reaction [1, 7, 8]. Unfortunately, it was also a time of tumultuous social and political change (for example, the ongoing war in Vietnam and the emergence of the “counterculture” movement). Anti-war sentiments, rising crime rates and the growing wave of drug addiction, psychedelic abuse, the sale of powerful hallucinogens (e.g., 2,5-dimethoxy-4-methylamphetamine, DOM, known as STP) as LSD on the black market and increasing numbers of hospital admissions related to the experience of “bad trips” in the United States of America and many other Western countries led to the stigmatization, vilification, and finally the criminalization of psychedelics in 1970 with the Controlled Substances Act (Schedule I). Following the restrictions introduced in the United States, strict drug control laws were introduced in other Western countries. These historical and political factors have severely limited scientific research into the clinical potential of psychedelic substances.

Recently, there has been renewed interest in psychedelics in the context of neuropharmacotherapy for mental illness. This is related to the growing global burden of mood and anxiety disorders and the emergence of promising clinical research results on psychedelic substances in the pharmacotherapy of depressive and anxiety disorders, in particular, post-traumatic stress disorder (PTSD), substance use disorders, and chronic pain [9–13]. Classical psychedelics have been shown to catalyze relatively long-lasting improvements in mental health after a small number of doses, especially when combined with psychotherapy [12]. Following the rapidly developing field of clinical research in psychedelic medicine, there have also been important changes at the regulatory level, with the decriminalization of natural psychedelics in Western countries (certain states in the USA, Canada, Australia, Switzerland, and the Netherlands) and the publication of guidelines for conducting research involving the use of psychedelics (U.S. Food and Drug Administration Guidance issued 20 June 1987; FDA-2023-D-1987) [14].

About the renaissance of psychedelics, apart from the clinical benefits, an analysis of the adverse effects and possible risks associated with their use in a medical context should be essential. In particular, we should focus on the pharmacological and molecular mechanisms and assess the cardiovascular safety associated with psychedelic therapy in randomized clinical trials. In the context of applied medical therapies, psychedelics are generally considered to be physiologically safe. They have low toxicity and low addictive potential [14, 15]. However, their agonism at serotonergic receptors should be investigated because of possible serotonin-related cardiotoxicity, influence on platelet aggregation, and proarrhythmic potential. The use of psychedelics has also been associated with significant sympathomimetic effects in both experimental and clinical studies [1, 16].

The present review aimed to undertake a critical discussion of the cardiovascular safety of classical psychedelics in pharmacotherapy based on pharmacological mechanisms as well as experimental and clinical research results. First, the cardiovascular effects of classical psychedelics in experimental studies were analyzed by searching the PubMed database. Then, documentation of clinical trials, observational studies of classical psychedelics was collected using the PubMed database. The searches were performed between July 1 and August 15, 2023, using the search terms psilocybin, mescaline, LSD, d-lysergic acid, ayahuasca, dimethyltryptamine, DMT and the clinical trials filter. Of these articles, only primary reports of classic psychedelics administered by a health professional alone were included. The presence of information on the observation and reporting of the presence or absence of cardiovascular effects after psychedelic treatment was analyzed in all retrieved articles and their supplementary materials (if available). The apparent adverse effects of this group of substances in terms of toxicity associated with non-medical use are, therefore, not addressed in this review.

## The mechanism of action of psychedelics

Classical psychedelics can be classified as tryptamines (e.g., psilocybin, its metabolite psilocin, N,N-dimethyltryptamine – DMT), phenethylamines (e.g., mescaline and its derivatives) and lysergamides (LSD) [9]. Experimental studies in the 1960s suggested that LSD activates the serotonergic system in the brain [1, 13, 17, 18]. The discovery of 5-HT receptor populations and the subsequent synthesis of selective 5-HT2 receptor antagonists enabled further development of knowledge on the mechanisms of the action of psychedelics. In particular, in a rat drug discrimination model study, Glennon et al. [19] were the first to show that blockade of 5-HT2 receptors with ketanserin and pirenperone abolished the effects produced by psychedelics. In support of this study, the binding of various hallucinogens to different populations of serotoninergic receptors using radioligand techniques indicated a common affinity only for 5-HT2, specifically 5-HT2A receptors [19–21].

Further animal and human studies have shown that different efficacy at 5-HT2A receptors is a key pharmacological mechanism responsible for the psychedelic effects [22–24] and that different psychedelic substances also have affinities for other receptors: serotonergic, dopaminergic, glutamatergic, adrenergic, and histaminergic [25–28]. Psychedelic stimulation of 5-HT2A receptors disrupts the cortical rhythm and large-scale brain networks through an increase in neocortical neuron excitation and subsequent augmentation of extracellular glutamate release in the prefrontal cortex [21, 29, 30]. In addition, psychedelics can affect neurotransmission by inhibiting transporters for the monoamine serotonin transporter (SERT) and vesicular monoamine transporter (VMAT2), trace amine-associated receptors (TAARs), and also monoamine oxidase (MAO) [31, 32]. Numerous experimental and human studies have confirmed the hypothesis of psychedelics as so-called ‘psychoplastogens’ as they were shown to enhance neurogenesis, neuroplasticity, and synaptic plasticity by increasing the expression of markers of neuroplastic processes (*c-fos Arc*) and brain-derived neurotrophic factor (BDNF) [33–36]. Recent animal studies suggest that the neuroplastic effects of psilocybin and LSD are associated with potent agonism (1000 times greater than classical antidepressants) at the tropomyosin-related kinase receptor B (TrkB) receptor for BDNF. This results in the enhancement of neurotrophic, plasticity and antidepressant-like behavior. It also promotes endogenous BDNF signaling [36].

## Mechanisms of the cardiovascular effects of psychedelics

**Fig. 1:**
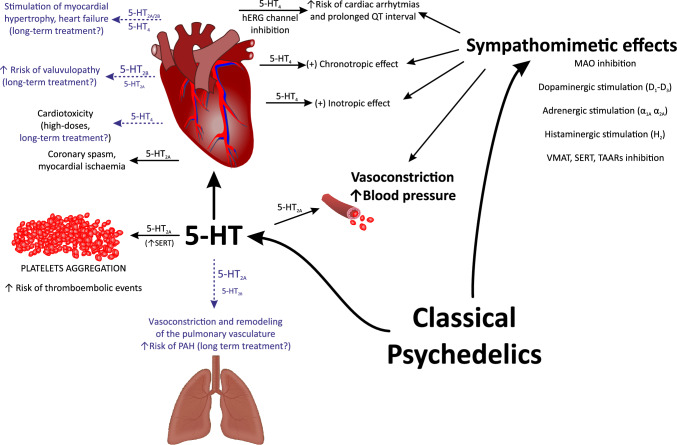
Cardiovascular effects of classical psychedelics according to experimental and clinical data (black text) and possible effects that need experimental and clinical investigation (blue text). *5-HT* serotonin; *MAO* monoamine oxidase; *PAH* pulmonary arterial hypertension; *SERT* serotonin transporter; *TAARs* trace amine-associated receptors; *VMAT* vesicular monoamine transporter

The effects of psychedelics on the cardiovascular system should be considered in terms of their effects on the 1) serotonergic system in the heart and the vasculature and/or their 2) sympathomimetic effects (Fig. 1).

### Cardiovascular effects of serotonin

Early experiments at the beginning of the twentieth century showed that blood platelets, as well as a substance isolated from enterochromatophilic cells (at that time called *enteramine*, later serotonin), act on smooth muscle by causing it to contract [37–39]. This substance was later identified as 5-hydroxytryptamine [39]. In adults, serotonin is mainly stored in dense platelet granules from which it is released during activation and aggregation phenomena, causing the vasoconstrictive effects described above. This circulating pool of 5-HT originates from the loading of platelets with serotonin synthesized in the intestinal wall. Serotonergic receptors are present in both the vascular endothelium (5-HT1B, 5-HT2B, and 5-HT7) and vascular smooth muscle (5-HT1B, 5-HT2A, and 5-HT7). Serotonin can produce both pressor and depressor effects. The diversity of vascular responses to 5-HT depends on the concentration, receptor site, and signaling mechanisms activated [40, 41]. In both humans and animals, 5-HT causes direct vasoconstriction predominantly via the 5-HT2A receptor and, to a lesser extent, via the 5-HT1B and 5-HT1D receptors [41, 42]. Serotonin also plays an important role in regulating coronary smooth muscle tone. Animal models and in vitro studies on the human coronary endothelium have shown that serotonin increases nitric oxide production, coronary vasodilation, and coronary flow via the 5-HT1B and 5-HT2B receptors [43–45]. Several observations provided evidence for increased vascular reactivity, i.e., a vasoconstrictor response to serotonin in damaged vessels (e.g., arterial hypertension). Namely, in an animal model of spontaneous hypertension (SHR), a six-fold and five-fold higher contractile sensitivity in the aorta and mesenteric artery, respectively [46], and an excessive central pressor response after exposure to 5-HT [47] have been demonstrated when compared to a normotensive control. The hypersensitivity to serotonin in experimental models of arterial hypertension may be related to enhanced expression of 5-HT2B serotonergic receptors in the smooth muscle of the vasculature [41, 48].

A large number of experimental studies and clinical observations have linked serotonergic hyperactivity to the development of pulmonary arterial hypertension (PAH). This is due to its vasoconstrictive and mitogenic effects on the vascular smooth muscle cells [49]. In addition, patients with PAH have been found to have high plasma serotonin concentrations [50]. Furthermore, both in humans and in experimental models of pulmonary hypertension, increased expression of the serotonergic receptors 5-HT2B and, to a lesser extent, 5-HT2A has been observed in pulmonary arteries [51–53]. There is also strong evidence that hypoxia alters protein expression and proliferative processes through mechanisms involving serotonin, its receptors, and its transporter. This leads to the physiological pulmonary responses to hypoxia, namely vasoconstriction and remodeling of the pulmonary vasculature [54].

Serotonin potentiates platelet aggregation via the 5-HT2A receptors. It promotes thrombosis and increases the risk of thrombotic events [55]. Serotonin 5-HT2A receptors are expressed on the membrane of human platelets, and their blockade by small drug-like molecules suppresses platelet aggregation induced by the extracellular release of 5-HT and P-selectin from platelets [56]. Further studies have provided evidence that 5-HT2A antagonists were effective in the inhibition of collagen-induced platelet aggregation and in offering protection against experimental thrombosis in animal models [57]. More recently, experimental studies have shown that sarpogrelate, a selective 5-HT2A receptor antagonist, prevented restenosis and thrombosis after stent implantation [58]. In addition, in patients with coronary artery disease, the expression of the 5-HT2A receptor was increased [59]. Interestingly, a very recent clinical study revealed that acute treatment with psychotropic drugs with 5-HT2A antagonist properties was associated with significantly lower mortality in elderly patients with severe COVID-19 infection. The mechanism of the protective effect of 5-HT2A receptor antagonists in COVID-19 is unknown but may involve potential immunomodulatory or antiplatelet effects [60].

Apart from the vasculature, serotonin receptors were found in the myocardium as well as in the valve apparatus of the heart. While two main populations of serotonin receptors, 5-HT2 (2A and 2B) and 5-HT4, have been described in the cardiac muscle (in both the atria and the ventricles), the expression of mainly 5-HT2B receptors has been described in cardiac valve tissue [40]. The serotonergic 5-HT2B receptors are expressed in heart valves from various species, including dogs, rats, pigs, monkeys, and humans [40, 61]. The messenger RNAs of the 5-HT1B and 5-HT1D receptor subtypes have been isolated from the human cardiac valve interstitial cells in only one study [62]. Serotonin increases contractility (positive inotropic effect), and reduces diastolic time (positive lusitropic effect) of the atria and ventricles of some mammals, including humans [40, 63, 64]. In addition, serotonin has been shown to increase heart rate (positive chronotropic effect) by activation of the sinus node cells in isolated preparations from humans and isolated samples from pig atria [40, 65, 66]. These effects were mediated by the 5-HT4 receptor and associated with an increase in cAMP (cyclic adenosine monophosphate) content and the subsequent elevation of the phosphorylation state of Ca^2+^ regulatory proteins [67]. Under normal conditions, stimulation of the 5-HT4 serotonin receptor did not produce significant effects due to the degradation of cAMP by phosphodiesterases. However, in the presence of a phosphodiesterase inhibitor, an effect of serotonin through 5-HT4 receptors has been demonstrated, leading to increased contractility, relaxation, and sometimes arrhythmias. These effects were reminiscent of those produced by catecholamines acting on beta-adrenergic receptors [68].

In the physiological state, the expression level of serotonin 5-HT4 receptors is low, but increases with ventricular dysfunction. Increased expression of the 5-HT4 receptor in the failing heart reflects a cardiac fetal reprogramming process [69, 70]. Cardiac expression of 5-HT2A and 5-HT2B receptors increases in hypertensive left ventricular hypertrophy and heart failure in humans and animals [70–72]. In mice, infusion of 5-HT2A antagonists reduced cardiac hypertrophy in a model of cardiac hypertrophy induced by transverse aortic constriction [72]. Therefore, the use of 5-HT2A/2B and 5-HT4 receptor stimulants in heart failure or myocardial hypertrophy should be considered given the possibility of the potentiation of serotonergic effects.

Serotonin has been implicated in pathological valve remodeling. The cardiac changes seen in the carcinoid syndrome, in which about 50 percent of the cases develop valvular lesions, are a clinical model of the effect of excessive serotonergic activity on the development of valvular lesions [61]. As mentioned above, the main serotonergic receptor on heart valve cells is the 5-HT2B receptor. The 5-HT2B receptor initiates several intracellular signaling cascades through both Gq proteins and β-arrestin. The 5-HT2B receptor also activates mitogenic pathways through Src kinase and extracellular regulated kinases (ERK), and enhances the activity of transforming growth factor β (TGF-β) [73]. In myofibroblasts and smooth muscle cells, the biological result of 5-HT2B receptor activation is mitosis, secretion of inflammatory cytokines, and extracellular matrix components [74, 75]. Huang et al. observed a proliferative response to 5-HT2B agonists in 5-HT2B-expressing HEK-293 cells [76]. Recently, in an animal model of transgenic mice, the overexpression of the Gq-coupled 5-HT2B receptor led to an increase in extracellular matrix, hypertrophic remodeling of the heart, and ultimately to reduced ventricular function [77]. In addition, blockade of the 5-HT2B receptor by cyproheptadine prevented pergolide-induced valvulopathy in rats [78].

### Cardiovascular effects of psychedelics in experimental studies

As agonists of serotonergic receptors, psychedelics have vasoconstrictive and, therefore, pressor effects. Dyer and Gant [79] reported that psilocin, psilocybin, mescaline, and LSD produced contractions of the isolated umbilical vasculature of humans and sheep, with LSD being the most potent. In another study, mescaline and LSD were found to induce cerebral vasospasm [80]. Apparently, the pressor effect after exposure to classical psychedelics depends on the type of substance used (the strongest effect in this group was shown for LSD) and, most importantly, on the dose. High doses of psychedelics has been responsible for dangerous reactions associated with severe vasospasm in different clinical case studies of drug intoxication in a non-medical context [1, 16, 81, 82]. This effect is exacerbated by the non-serotonergic effects of psychedelics, such as interaction with monoaminergic targets (adrenergic, dopaminergic, histaminergic, MAO, monoamine transport) [25–28, 31, 32].

As mentioned above, serotonergic hyperactivity is associated with an increased risk of the development of pulmonary arterial hypertension. However, information on the effects of psychoactive substances on pulmonary arterial pressure or in animal models of pulmonary hypertension is lacking in the available literature. Given the role of serotonin in the above pathophysiological mechanisms of PAH, it seems reasonable to investigate the effects of psychedelics on pulmonary pressure in chronic use and to assess the safety of their use in patients with pulmonary hypertension and/or chronic hypoxia.

Serotonin increases platelet aggregation via 5-HT2A receptors. This raises the question of whether psychedelics increase the risk of thromboembolic complications. Hallucinogens such as LSD, DMT, and mescaline have been found to induce the human thrombocyte shape-changing response [83]. However, serious thromboembolic events associated with psychedelic use have only been described in situations of reactive drug abuse [82, 84, 85].

Experimental studies on the effects of psychedelics on heart function provided inconsistent information. In endothelin-1-induced hypertrophy and tumor necrosis factor-α (TNF-α)-induced cell injury in H9C2 cardiomyocytes, water extracts of *Panaeolus cyanescens* and *Psilocybe cubensis* did not aggravate the pathological hypertrophy induced by endothelin-1 and also protected against the TNF-α-induced injury and cell death. Specifically, psilocybin-containing mushroom extracts have been shown to reduce the size of cardiomyoblasts, increase mitochondrial activity, and reduce TNF-α and oxygen-free radical levels [86]. Recently, Gergs et al. [87] demonstrated the inotropic and chronotropic effects of LSD via the serotonin 5-HT4 and histamine H2 receptors. In this study, LSD in a dose-dependent manner increased contractility and beating rates in spontaneously beating right atrial preparations and in spontaneously beating Langendorff-perfused hearts from transgenic mice overexpressing the human 5-HT4 receptor, the H2-histamine receptor, and also in the human right atrial muscle. These effects were antagonized by H2-histamine and 5-HT4 antagonists. In another study, psilocin administration to Wistar rats by intraperitoneal injection for 12 weeks produced ECG abnormalities in the form of tachycardia, myocardial ischaemia, abnormal intraventricular conduction and mitochondrial degeneration, and myocardial purine profile changes [88]. Moreover, psilocybin intoxication induced systolic dysfunction and takotsubo cardiomyopathy, possibly due to its toxic effect on the cardiomyocytes or strong sympathomimetic effect and interaction with not only serotonergic but also adrenergic and dopaminergic receptors [89, 90]. Consistent with these studies, in an animal model and the H9C2 cell lines, the use of the phenethylamines, 2-((2-(4-Iodo-2,5-dimethoxyphenyl)ethylamino)methyl)phenol (25I-NBOH) and 2-(((2-(4-chloro-2,5-dimethoxyphenyl)ethyl)amino)methyl)phenol (25C-NBOH), dramatically reduced the viability of the H9C2 cardiomyocytes and downregulated p21 CDC42/RAC1-activated kinase 1 (PAK1). In addition, 25I-NBOH demonstrated a proarrhythmic potential as it was shown to inhibit the potassium channels in the human ether-a-go-go-related gene (*hERG*) assay and to significantly prolong the QT intervals and RR intervals in the rat ECG measurement [91]. The risk of the proarrhythmic effects of psilocin was also investigated in another experimental study. In tsA201 cells, psilocin reduced the hERG currents in a concentration-dependent manner. However, in this observation, a 500-fold higher concentration of psilocin than the concentration actually achieved in human plasma in clinical studies was required to produce a relevant inhibition of the hERG channel [92]. Thus, the proarrhythmic effect of psilocybin, resulting in a significant prolongation of the QT interval, appears to occur at high doses or to depend on mechanisms other than inhibiting the hERG current.

Psychedelic drugs may increase the risk of heart valve disease by binding to the 5-HT2B receptors. This risk should be considered both in terms of prolonged exposure to substance used and the affinity of the psychedelic for the 5-HT2B receptor itself. Surprisingly, neither experimental nor clinical data have comprehensively assessed this risk. The only experimental studies that have included a histological evaluation of the heart have been conducted with ayahuasca – a hallucinogenic beverage containing the beta-carbolines (harmine, harmaline, and tetrahydroharmine) and N,N-dimethyltryptamine [93, 94]. In the first study, no abnormalities were evident in a histological examination of the cardiac tissues of Wistar rats carried out 14 days after the administration of ayahuasca at a dose 50 times higher than in religious ceremonies (15.1 mg/kg DMT) [93]. A second animal study, in which ayahuasca was administered daily for 28 days in doses that exceeded those typically used in religious ceremonies, also showed that there were no histopathological changes in the heart [94]. The results of these experimental studies must be treated with some caution because ayahuasca is a mixture of DMT and beta-carbolines, which have different effects on the cardiovascular system. Indeed, in cardiovascular research, harmine has been shown to reduce systemic arterial blood pressure and peripheral vascular resistance through the inhibition of L-type calcium voltage-dependent channels. Finally, a recent study provided several lines of evidence for the anti-hypertrophic effects of harmine. Namely, in an animal model of spontaneous hypertension (SHR), harmine reduced myocardial hypertrophy. In addition, in vitro observations (of human embryonic stem cell-derived cardiomyocytes) showed that by inhibiting NF-κB phosphorylation and reducing inflammation, harmine inhibited the phenotypes of norepinephrine-induced hypertrophy and also downregulated the expression of hypertrophy-related genes [95]. There are no experimental studies on the risk of valve injury associated with the prolonged use of psilocybin and LSD. This contrasts with the other hallucinogen 3,4-methylenedioxymethamphetamine (MDMA, ‘Ecstasy’), for which prolonged mitogenic effects and induction of valvular interstitial fibroplasia through activation of the 5-HT2B receptors have been demonstrated [96]. In the absence of sufficient in vivo data, we must rely on in vitro data to assess the risk of valvular heart disease associated with medical psychedelic therapies (particularly microdosing with LSD and psilocybin). Psilocybin is a prodrug as it is almost completely metabolized to its active metabolite psilocin during absorption. Holze et al. [97] showed that the geometric mean plasma Cmax of 15 mg psilocybin was 13 ng/ml psilocin (63.5 nM). Considering psilocybin/psilocin dose proportional exposure, a typical microdoses of 1 and 2 mg of psilocin would have a Cmax of 4.2 and 8.4 nM, respectively. For LSD, pharmacokinetic studies following a 100 µg dose have reported a geometric mean Cmax of 0.279 ng/ml (0.86 nM) [98]. The concentrations of the two major metabolites of LSD [2-oxo-3-hydroxy-LSD and N-demethyl-LSD] used in in vitro studies at the 100 µg dose were very low and showed very low affinity for the 5HT-2B receptor. Therefore, in theory, the toxic effects of LSD metabolites at the LSD doses normally used for microdosing seem unlikely [99]. Pharmacokinetic data (Table 1) indicate that psilocybin/psilocin has a high potency for the 5-HT2B receptor, whereas in the case of LSD, the affinity for the 5-HT2B receptor is similar to its affinity for the 5-HT2A receptor. This suggests that psilocybin may have a relatively higher risk of valvular heart disease than LSD in microdosing therapies [100].

**Table 1:** Binding affinity (Ki) and potency (EC50) of psilocin and d-lysergic acid (LSD) at 5-HT2A and 5-HT2B receptors

References	5-HT2A		5-HT2B	
	Ki (Radiologand)	EC_50_[*E*_max_]^a^ (Assay)	Ki (Radiologand)	EC_50_[*E*_max_]^a^ (Assay)
Psilocin				
Glatfelter et al. [101]	180 ([^3^H]-ketanserin)	13 [67%] (Ca)	8 ([^3^H]-LSD)	8 [38%] (Ca)
		81 [76%] (β-arrestin2)		34 [84%] (β-arrestin2)
Halberstadt and Geyer [102]	107.2 ([^3^H]-ketanserin)		4.6 ([^3^H]-LSD)	
Klein et al. [103]		2.4 [98.4%] (Ca)		2.4 [39%] (Ca)
Ray et al. [27]	339.6 ([^3^H]-LSD)		4.7 ([^3^H]-LSD)	
Rickli et al. [104]	49 ([^3^H]-ketanserin)	721 [16%] (Ca)		> 20,000 [not determined] (Ca)
Sard et al. [105]		24 [43%] (PI)		58 [45%] (PI)
d-lysergic acid				
Luethi et al. [99]	3.1 ([^3^H]-ketanserin)	225 [60%] (Ca)		207 [13%] (Ca)
Nichols et al. [106]	3.5 ([^125^I]-DOI)	15 [23%] (PI)	30 ([^125^I]-DOI)	
Porter et al. [107]		21.4 [44%] (Ca)		8.9 [44%] (Ca)
Ray et al. [27]	11.3 ([^3^H]-LSD)		30 ([^3^H]-LSD)	
Rickli et al. [104, 108]	4.2 ([^3^H]-ketanserin)	261 [28%] (Ca)		12,000 [71%] (Ca)

Values given in nM. Activation efficacy (Emax) is relative to serotonin

*Ca* intracellular calcium assay; *G*_*q*_ G_q_ dissociation assay, *PI* phosphoinositol hydrolysis assay

At this point it should also be noted that the development of experimental models (mainly in rodents) of psychedelics-associated valvulopathies and their translation to human studies is difficult, possibly because of interspecies differences in response to valvulotoxic agents or sensitivity to induction of valvular defects. Heart rate and local hemodynamic pressures during the cardiac cycle vary considerably depending on the body weight of the species. An example of this is the development of an animal model of heart valve damage caused by the drugs fenfluramine and phentermine (Fen-Phen). Despite the demonstration of serotonin receptor-mediated degenerative changes induced by Fen-Phen administration in vitro in cultured valve interstitial and endothelial cells and in humans, replication of these changes in an in vivo model has had limited success. In one study, Bratter et al. administered a continuous subcutaneous infusion of Fen-Phen to pregnant rats at a dose approximately 10 times the clinical dose for humans. As a result, large lesions of the mitral valve were observed in 25 percent of the pups [109].

The effects of psychedelics on the cardiovascular system such as the pressor and tachycardic response are also related to their sympathomimetic mechanism [110]. As mentioned earlier, psychedelics have been shown to interact with other than serotonergic monoaminergic targets, i.e., adrenergic receptors (α2A and α1A), dopaminergic receptors (D1-3), histaminergic receptors, TAARs1, monoamine transporters, and MAO inhibition.

Observations suggesting effects of psychedelics (psilocybin and LSD) on the TrkB/BDNF pathway may also be relevant to potential cardiovascular effects of psychedelics [36]. Many tissues and non-neuronal cells can synthesize BDNF. These include myocardium, endothelium and platelets [111]. In the adult heart, an impairment of the BDNF-TrkB pathway has been linked to reduced cardiac function, myocyte death, increased cardiac inflammation and oxidative stress, leading to the development of HF. [112, 113]. Through the promotion of TrkB/BDNF signaling, LSD and psilocybin may also have possible effects on cardiomyocytes and vascular endothelial function. Research into the mechanism of action of these drugs would be of interest.

## Evidence-based cardiovascular risk related to psychedelic therapy

Assessing the cardiovascular safety of psychedelics in pharmacotherapy is challenging because the efficacy and potential adverse effects of these substances depend on complex factors such as the type of substance used, its dose, and, arguably, the duration of therapy. A patient’s comorbidities and drug–drug interactions must also be taken into account. There is also a need for a differentiated assessment of the potential risk of psychedelic use in relation to the potential benefits that patients may derive from the therapy. In randomized controlled trials (RCTs), observational data studies, and systematic reviews, psychedelics have been shown to produce short-term and clinically non-significant sympathomimetic effects, including increased heart rate and blood pressure. This effect was found to be dose dependent for all the classical psychedelics.

However, the remote effects of psychedelics have not been investigated in any of these studies. An interesting study on the possible long-term effects of psychedelics has recently been published. Namely, Simonnson et al. [114] showed that people who reported at least one-lifetime use of classical psychedelics (for non-medical purposes) had significantly lower odds of hypertension. It is worth noting that when the associations between hypertension in the past year and lifetime use of the main classes of classical psychedelics were analyzed, only the association with lifetime use of tryptamines was significant.

### Psilocybin

An analysis of the clinical data on psilocybin suggests that it is relatively safe at the doses that have been used (Table 2). However, some published studies, especially those in which psilocybin was administered over a longer period (2–3 weeks in the microdose regimen), do not mention cardiovascular monitoring and cardiovascular effects [115–119]. The most commonly observed adverse effects following psilocybin were increased heart rate and blood pressure. These changes were transient, dose dependent and there was no need for medical intervention. A more serious adverse effect after psilocybin administration was a prolongation of the QTc interval [120]. In many studies, ECGs were performed only at enrolment, and electrocardiographic screening was not performed, so the magnitude of the effect of psilocybin on QT interval prolongation may be greater. Given the risk of serious life-threatening arrhythmias, electrocardiographic monitoring should be a *conditio sine qua non* not only in studies with psilocybin but also in studies with other psychedelics. Also, in view of the risk of valvulopathy associated with the pharmacodynamics of psilocybin (strong agonism at the 5-HT2B receptor), its chronic use in the ‘microdose’ form requires echocardiographic monitoring.

**Table 2:** Cardiovascular effects of psilocybin in clinical trials

References	Year	Number of participants and dosage	Cardiovascular effects reported
Bogenschutz et al. [121]	2015	10 volunteers with alcohol dependence received orally administered psilocybin 0.3 mg/kg	No significant treatment-related cardiovascular adverse events were reported
Brown et al. [122]	2017	12 healthy volunteers were given psilocybin at a dose of 0.3, 0.45, or 0.6 mg/kg	The side effects included mild transient hypertension and tachycardia
Carhart-Harris et al. [123]	2016	12 patients with major depression received two doses of psilocybin (10 and 25 mg, 7 days apart)	No significant treatment-related cardiovascular adverse events were reported
Carhart-Harris et al. [124]	2021	59 patients with major depressive disorder received 25 mg or 1 mg psilocybin, 3 weeks apart	No significant treatment-related cardiovascular adverse events were reported. Heart palpitations occurred with similar frequency as after escitalopram (10.3%)
Carbonaro et al. [125]	2018	20 healthy volunteers received psilocybin at a dose 10, 20, or 30 mg/70 kg	The authors reported that blood pressure increased to 138/80 mmHg after 10 mg/70 kg psilocybin, 142/85 mmHg after 20 mg/70 kg, and 140/87 mmHg after 30 mg/70 kg. 30 mg/kg psilocybin increased HR to 94 beats/min
Dahmane et al. [126]	2021	Assessment of the proarrhythmic potential of psilocybin (QTc analysis) in relation to psilocybin at oral doses of 0.3 mg/kg (*n = *12), 0.45 mg/kg (*n = *11), and 0.6 mg/kg (*n = *10). Each dosing period was separated by 4 weeks	Only high doses of psilocybin (42–59 mg) caused significant QTc prolongation. The standard dose of 25 mg has been shown to be relatively safe
Davis et al. [127]	2021	24 volunteers with major depressive disorder received psilocybin at two doses in two sessions (session 1: 20 mg/70 kg; session 2: 30 mg/70 kg)	Mean peak heart rate and blood pressure values were 86.6 (± 16.3) beats/min and 138.7 (± 13.0)/87.6 (± 9.1) mmHg during session 1 and 86.6 (± 13.1) beats/min and 140 (± 10.7)/87.1 (± 7.2) mmHg during session 2
Goodwin et al. [120, 128]	2022, 2023	233 patients with treatment-resistant episode of major depression received psilocybin at doses 25 mg (*n = *79), 10 mg (*n = *75), 1 mg (*n = *79) 1 mg	Psilocybin induced a change from baseline in QTc > 60 ms on day 2 in two cases in the 25 mg dose group
Griffiths et al. [129]	2016	51 cancer patients randomized to psilocybin therapy at low-dose (1 or 3 mg/70 kg) and high-dose (22 or 30 mg/70 kg) in a counterbalanced sequence with 5 weeks between sessions and a 6-month follow-up	Mean peak heart rate and blood pressure values were 78.9 (± 2.2) beats/min and 142.2 (± 2.5)/82.9 (± 1.4) mmHg during low-dose session and 84.1 (± 2.6) beats/min and 155 (± 2.9)/89.7 (± 1.2) mmHg during high-dose session. 34% of the participants in the high-dose session and 17% of the participants in the low-dose session experienced an episode of elevated systolic blood pressure (greater than 160 mmHg). An episode of elevated diastolic blood pressure (greater than 100 mmHg) occurred in 13% of those receiving the high dose and 2% of those receiving the low dose
Grob et al. [130]	2011	12 patients with advanced-stage cancer and anxiety received a moderate dose (0.2 mg/kg) of psilocybin	Heart rate and blood pressure peaked at 81.5 (± 5.8) beats/min and 138.9/75.9 mmHg 2 h after psilocybin compared to 70.4 (± 4.3) beats/min and 117/69.6 mmHg when taking placebo. No sustained tachyarrhythmias or heart block were observed on Holter monitor recordings during the psilocybin sessions
Gukasyan et al. [131]	2022	27 patients with unipolar depression received 2 doses of psilocybin at 20 mg/70 kg and 30 mg/70 kg, approximately 2 weeks apart	No information about cardiovascular parameters screening in this study
Hasler et al. [132]	2004	8 healthy volunteers received a very low dose of 45 g/kg body weight, a low dose of 115 g/kg, a medium dose of 215 g/kg, and a high dose of 315 g/kg of psilocybin	Mean arterial pressure and heart rate increased 60 min after high-dose psilocybin (placebo 93 ± 3.9 mmHg and 78 ± 5 beats/min, low-dose psilocybin 97 ± 3.9 mmHg and 79 ± 5 beats/min, high-dose psilocybin 101 ± 3.9 mmHg and 72 ± 6 beats/min). No ECG changes were reported
Holze et al. [97]	2022	28 healthy subjects received psilocybin (15 and 30 mg) and d-lysergic acid diethylamide (LSD) (100 and 200 µg)	Blood pressure (BP) was increased by both LSD and psilocybin. Psilocybin increased BP more than LSD, whereas LSD increased heart rate more than psilocybin. Compared with LSD (138/86 and 141/87 mmHg for 100 and 200 µg, respectively) and 15 mg psilocybin (140/89 mmHg), 30 mg psilocybin produced significantly greater increases in BP (146/93 mmHg). Psilocybin moderately increased heart rate compared with the placebo (74 beats/min) at 30 mg (82 beats/min)
Johnson et al. [133]	2014	15 nicotine-dependent smokers received moderate (20 mg/70 kg) and high (30 mg/70 kg) doses of psilocybin	Blood pressure and heart rate were elevated during the drug effect. Systolic blood pressure showed a mean peak value of 153 mmHg (compared with the baseline: 125 mmHg). Diastolic blood pressure showed a mean peak of 87 mmHg (compared with the baseline: 71 mmHg). Heart rate showed a mean peak of 87 beats/min (compared with the baseline: 68 beats/min)
Mallaroni et al. [134]	2023	11 subjects received 15 mg of psilocybin	Significant pressor effects were produced by psilocybin (121.6/76.9 vs. 112.5/68.6 mmHg). In 8 cases, systolic hypertension (greater than 140 mmHg) was observed
Moreno et al. [135]	2006	9 participants with obsessive–compulsive disorder received low (100 µg/kg), medium (200 µg/kg), and high (300 µg/kg) doses of psilocybin	One subject experienced transient hypertension
Rucker et al. [136]	2022	89 healthy participants received a dose of 10 or 25 mg psilocybin	No cardiovascular effects were reported
Schindler et al. [137]	2021	10 adults with migraine received an oral placebo and psilocybin (0.143 mg/kg) in 2 test sessions spaced 2 weeks apart	Post hoc analysis revealed a significant increase in mean arterial pressure with psilocybin administration from 45 min to 4 h after ingestion. The max. increase in mean arterial pressure compared with the placebo was 12.2 (4.61–19.73) mmHg at 1.5 h post-ingestion
Schneier et al. [138]	2023	12 adults received psilocybin at a dose of 25 mg	Mild increase in heart rate (69 vs. 74 beats/min) and no change in blood pressure were reported (118.75/76 vs. 117/72 mmHg)

### Mescaline

Data from clinical trials on the therapeutic use of mescaline are scarce. A recent study compared the immediate acute subjective, cognitive, and cardiovascular effects of 2,5-dimethoxy-4-bromophenethylamine (2C-B), a mescaline-derived hallucinogenic phenethylamine, with psilocybin in a group of 11 women [134]. The participants were given 20 mg of 2C-B, 15 mg of psilocybin, or a placebo on three separate occasions. Both compounds induced pressor effects (systolic and diastolic blood pressure). Systolic hypertension (greater than 140 mmHg) was observed in 5 cases with 2C-B and 8 cases with psilocybin. No significant differences in the heart rate of patients were observed. Recently, Ley et al. [139] compared the acute effects of mescaline (300 mg and 500 mg), LSD (100 µg), and psilocybin (20 mg) in 32 healthy subjects. Systolic blood pressure and heart rate were similarly increased by the high dose of mescaline (500 mg), LSD, and psilocybin. Notably, the increase in heart rate in response to the low dose of 300 mg mescaline exceeded the increase in heart rate in response to the high dose of 500 mg mescaline. When heart rate and blood pressure increases were combined using the rate-pressure product, the overall cardiovascular stimulation was comparable for all three substances.

### N,N-dimethyltryptamine (DMT) or ayahuasca

Clinical trials using N,N-dimethyltryptamine have included DMT alone and ayahuasca, which contains DMT. In studies where cardiovascular effects were reported, administration of DMT or ayahuasca had transient, non-clinically significant sympathomimetic effects (tachycardia or an increase in blood pressure) (Table 3). When analyzing the results of these studies, it is important to remember that ayahuasca contains many other potentiating ingredients in addition to DMT. These include MAO inhibitors such as harmaline, which may affect depression (and the results of the studies) independently of DMT.

**Table 3:** Cardiovascular effects of N,N-dimethyltryptamine (DMT) or ayahuasca in human clinical studies

References	Year	Number of participants and dosage	Cardiovascular effects reported
Dos Santos et al. [140]	2012	Ayahuasca was administered in 3 sessions to 17 healthy volunteers at a dose equivalent to 0.75 mg DMT/kg body weight	Systolic blood pressure increased above 140 mmHg in 5 cases. Diastolic blood pressure values did not reach values above 90 mmHg for any participant. Tachycardia was reported in 1 case
D’Souza et al. [141]	2022	Healthy volunteers (*n = *3) and patients with major depressive disorder (*n = *7) received DMT in two doses 0.1 mg/kg and 0.3 mg/kg	DMT increased blood pressure and heart rate. Peak values for blood pressure and heart rate were 144/84 mmHg and 75 beats/min at the 0.1 mg/kg DMT dose and 147/80 mmHg and 96 beats/min at the 0.3 mg/kg DMT dose). At 0.3 mg/kg, one serious adverse event occurred in a subject with asymptomatic bradycardia and hypotension
Durante et al. [142]	2021	614 participants answered an online questionnaire about its safety	Tachycardia was reported by 200 (32.57%) participants as occasionally occurring, 47 (7.67%) participants as frequently occurring, 10 (1.63%) participants as always occurring, and 9 (1.47%) participants as persistently occurring
Riba et al. [143]	2002	18 volunteers received encapsulated freeze-dried ayahuasca (0.6 and 0.85 mg of DMT/kg)	Diastolic blood pressure (DBP) increased at the high dose (Δ DBP 9 mmHg). Systolic blood pressure and heart rate did not increase
Strassman et al. [144]	1994	11 experienced hallucinogen users received DMT iv at doses of 0.05, 0.1, 0.2,0.4 mg/kg	DMT increased blood pressure and heart rate in a dose-dependent manner
Vogt et al. [145]	2023	27 healthy participants received different regimens of intravenous DMT: placebo, low or high infusion (0.6 or 1 mg/min), low bolus + low infusion (15 mg + 0.6 mg/min) and high bolus + high infusion (25 mg + 1 mg/min)	Bolus doses of DMT produced rapid, marked increases in blood pressure (peak systolic and diastolic pressures 159 ± 3.4 mmHg and 98 ± 1.6 mmHg, respectively) and heart rate (max 119 ± 4.2 bpm), peaking within 2 min

### d-lysergic acid diethylamide (LSD)

Like psilocybin, LSD has shown no significant cardiovascular adverse effects in studies primarily involving healthy volunteers (Table 4). A pressor response and an increase in heart rate were the most common dose-dependent cardiovascular effects observed in these clinical trials. LSD increased blood pressure and heart rate in a dose-dependent manner. It is worth noting, however, that the cardiovascular response to LSD tends to differ. Holze et al. showed that LSD at doses of 100 and 200 µg accelerated the heart rate more than psilocybin (at doses of 15 mg and 30 mg) and that psilocybin increased blood pressure more than LSD [97]. Similar changes in the cardiovascular system after identical doses of LSD have been reported by Dodler [146]. Whereas Gasser [147] reported that LSD at a dose of 200 µg had no effect on blood pressure or heart rate. In other studies, much lower doses of LSD (below 26 µg) induced a pressor response and tachycardia [148, 149]. In another case, no changes in cardiovascular haemodynamic parameters were observed after administration [150], even in a healthy elderly population [151]. Perhaps these differences are related to the heterogeneous patterns of LSD use (Table 4) or to the study population itself (healthy volunteers with no history of reactive use of psychedelics versus those who reported a history of psychedelic use). Another explanation could be the different pharmacokinetics following the administration of LSD, as a large variability in blood concentrations of LSD has been found after microdosing [151].

**Table 4:** Cardiovascular effects of d-lysergic acid diethylamide (LSD) in human clinical studies

References	Year	Number of participants and dosage	Cardiovascular effects reported
Bershad et al. 2019 [148]	2019	20 healthy adults attended four laboratory sessions during which they received a placebo, 6.5 µg, 13 µg, or 26 µg of LSD in random order at weekly intervals	LSD increased systolic blood pressure from 105.35 mmHg in the placebo session to a peak of 111.5 at 13 µg and 115.3 at 26 µg, and 26 µg increased diastolic blood pressure. The drug had no significant effect on heart rate
Bershad et al. [149]	2020	20 healthy adults participated in four sessions. They received a single low dose of LSD (13 µg) or a placebo	LSD was associated with a significant increase in systolic blood pressure (108.9 vs 114.4 mmHg)
de Wit et al. [150]	2022	56 healthy participants were assigned to one of three drug conditions: placebo,13 µg or 26 µg of LSD	LSD had no significant effect on heart rate or blood pressure during any of the sessions
Dodler et al. [146]	2016	40 volunteers received LSD at doses of 100 µg (24 subjects) and 200 µg (16 subjects)	At both doses, LSD caused similar increases in diastolic and systolic blood pressure and heart rate
Family et al. [151]	2020	A total of 48 elderly volunteers were randomly assigned to 1 of 4 LSD dose groups (5, 10, and 20 μg LSD, and placebo). They received their assigned dose six times (i.e., every 4 days)	LSD was well tolerated and the incidence and severity of adverse events were similar to the placebo. No clinically significant abnormalities were reported based on physical examinations and ECG measurements
Gasser et al. [147]	2014	12 patients with anxiety associated with a life-threatening illness took part in an LSD-supported psychotherapy trial. The participants received either 200 µg of LSD or 20 µg of LSD with an open-label crossover to 200 µg of LSD after the initial blinded treatment had been unmasked	LSD had no significant effect on blood pressure or heart rate
Holze et al. [97]	2022	28 healthy subjects underwent five 25 h sessions and received a placebo, psilocybin (15 mg and 30 mg) or LSD (100 and 200 µg)	For details see also Table 1 Both doses of LSD increased heart rate more than both doses of psilocybin and placebo (peak heart rate 74 ± 2.0 beats/min for placebo; 78 ± 2.1 and 82 ± 3.1 beats/min for 15 and 30 mg psilocybin and 83 ± 25 beats/min and 90 ± 3.2 for 100 and 200 µg LSD)
Holze et al. [98]	2021	16 healthy volunteers received a placebo, LSD (25, 50, 100, and 200 µg) and 200 µg LSD 1 h after administration of the 5-HT2A antagonist ketanserin (40 mg) during six sessions (25 h each)	LSD increased blood pressure at doses of 50 µg or higher (peak values for placebo 131 ± 3.5/80 ± 2.1 mmHg and 200 µg LSD 138 ± 2.4/87 ± 2.0 mmHg). Heart rate also increased at 100 and 200 µg LSD doses (83 ± 3.3 and 86 ± 3.9 beats/min vs 75 ± 2.7 beats/min for placebo). Ketanserin prevented the heart rate response induced by LSD and transiently reduced the blood pressure response induced by LSD
Holze et al. [152]	2022	A pooled analysis of four double-blind, randomized, placebo-controlled, crossover studies involving 83 healthy volunteers who received LSD at single doses of 25, 50, 100 and 200 µg	Doses above 25 µg produced significant acute and transient increases in blood pressure and heart rate. A dose-dependent effect was observed for increases in heart rate but not blood pressure. Systolic blood pressure > 140, > 160 and > 180 mmHg were observed in 48%, 5% and 0% of all LSD doses, respectively. The maximum values for diastolic and systolic blood pressure were 103 and 173 mmHg, respectively Tachycardia was observed in 15% of all LSD doses. The maximum heart rate was 129 beats/min
Murray et al. [153]	2022	22 healthy men and women underwent three EEG sessions. Under double-blind conditions, they received a placebo or LSD (13 µg and 26 µg)	LSD increased heart rate and both systolic and diastolic blood pressure
Olbrich et al. [154]	2021	25 healthy volunteers were divided into three groups: placebo + placebo; placebo + LSD (100 µg); LSD + ketanserin (40 mg)	The psychedelic effects of LSD were positively associated with sympathetic activity and negatively associated with parasympathetic activity. LSD treatment increased heart rate. Ketanserin decreased heart rate compared with the placebo
Ramaekers et al. [155]	2020	24 healthy volunteers received single doses of 5, 10, and 20 µg of LSD and placebo on separate occasions	LSD treatment affected both systolic and diastolic blood pressure. Separate contrasts showed that LSD at 10 µg increased the diastolic blood pressure, whereas LSD at 20 µg increased both the systolic and the diastolic blood pressure. LSD treatment did not affect heart rate
Schmid et al. [23]	2015	LSD (200 µg) and a placebo were administered to 16 healthy volunteers in a double-blind, randomized, placebo-controlled, crossover study	LSD increased systolic and diastolic blood pressure and heart rate. The maximum values for systolic and diastolic blood pressure and heart rate were 148.4 ± 3.0 mmHg, 87.6 ± 1.9 mmHg, and 87.9 ± 4.3 beats/min, respectively

As with psilocybin, many papers do not report changes in cardiovascular parameters during the use of LSD [156], or the authors simply monitored baseline BP and heart rate without assessing them after LSD administration [157].

## The safety of the cardiovascular system during psychedelic therapy—what more do we need to know? What else should be done?

The physiological safety of single doses of psychedelics is now relatively well established. However, the potential risks of prolonged low-dose administration of psychedelics, particularly in relation to the cardiovascular system, require further research. Despite the growing evidence for the potential benefits of classical psychedelics in treating psychiatric disorders and the relative cardiovascular safety of psychedelic-assisted therapies, it should be remembered that, based on previous experience, prolonged pharmacological induction of serotonergic 5-HT2B receptor activation can lead to serious cardiac complications. Several drugs with relatively high 5-HT2B receptor binding affinity (Ki < 500 nM) have been unequivocally associated with valvular heart diseases, including fenfluramine and dexfenfluramine, methylergonovine, methysergide, ergotamine, pergolide, and cabergoline. Approximately 25 percent of patients developed new onset valvulopathy, including some cases of valvular thickening leading to death or requiring heart surgery [158, 159]. For this reason, it is necessary to include echocardiographic monitoring during and after prolonged use of psychedelics.

Clinical trials with psychedelics in the form of microdosing (mainly LSD and psilocybin) have not provided convincing evidence, and the potential risks may outweigh the benefits of the treatment. More convincing evidence and rigorous placebo-controlled clinical trials using representative populations are needed to establish medical indications for the chronic use of psychedelics. The largest “self-blinding” clinical trial to date, with 191 participants who received an average of 13 µg of LSD or 0.2 g of psilocybin mushrooms microdosed every 3–4 days for 4 weeks, showed equal improvement in both the placebo and psilocybin groups. This suggests that the beneficial effects of psychedelics could be explained by the placebo response [160]. In a recently published randomized clinical trial with groups assigned to placebo, 13 µg LSD, or 26 µg LSD, low doses of LSD were shown to be safe but produced negligible changes in mood or cognition in healthy volunteers [150]. It is also important to develop consistent, similar regimens for the use of microdosing therapies and to define the maximum duration of therapy.

Finally, existing clinical trials using psychedelics in a medical context have excluded patients with cardiovascular disease. Given the lack of evidence in this area, the relative safety of classical psychedelics does not apply to cardiovascular patients.

Another important issue that should be the subject of specific experimental and clinical studies is the assessment of interactions between psychedelics and their metabolites and other therapies. Given that psychedelics affect serotonergic neurotransmission, there is a potential for drug-drug interactions with medications that also modulate the serotonin system such as selective serotonin and serotonin-norepinephrine reuptake inhibitors, tricyclic antidepressants, monoamine oxidase inhibitors, atypical antipsychotics, and others. Some of these interactions may increase the risk of serotonin toxicity and even the development of symptoms of life-threatening serotonergic syndrome [161, 162]. Nevertheless, some clinical cases have shown that the chronic administration of the serotonin transporter inhibitors fluoxetine and sertraline reduced the effects of LSD and psilocybin in recreational drug users [163, 164]. This is supported by the results of a clinical study investigating whether escitalopram pretreatment for 2 weeks enhances the acute response to 25 mg of psilocybin in a group of 23 healthy volunteers. In this study, escitalopram did not have a relevant effect on the positive mood effects of psilocybin, but significantly reduced bad drug effects, anxiety, adverse cardiovascular effects and other adverse effects of psilocybin compared with placebo. In addition, the pharmacokinetics of psilocin were not altered by escitalopram [165]. Liver enzyme polymorphisms and the co-medication of patients undergoing psychedelic-assisted psychotherapy may potentially influence the pharmacology of psychedelics, and the chronic use of psychedelics may influence the activity of other drugs. Luethi et al. provided evidence for LSD metabolism in human liver microsomes (i.e., CYP2D6, 1A2, 3A4, and 2E1). Therefore, there is a possibility of drug–drug interaction with cardiovascular drugs such as statins (atorvastatin and lovastatin), warfarin, or ticagrelor [99].

## Summary

The resurgence of scientific interest in psychedelic medicine has led to new insights into an ancient class of pharmacological substances used by humans for ritual, therapeutic, and cultural reasons. As this field of research evolves, there may be a need for an update of knowledge about psychedelic drugs. For patients who do not benefit from currently available treatments, continued medical research and scientific exploration of psychedelic drugs may offer new ways to treat mental illness and addiction. However, there are still many unknowns that need to be addressed to determine the safety and minimize the risk of psychedelic pharmacotherapy. Cardiovascular safety is of particular concern because of potential serotonin-related cardiotoxicity. The exact determination of the cardiovascular safety of psychedelic therapies (especially long-term therapies) requires further research.

## Data Availability

Data sharing is not applicable to this article as no datasets were generated or analyzed during the current study.
